# Common Genetic Variants of the Human Steroid 21-Hydroxylase Gene (*CYP21A2*) Are Related to Differences in Circulating Hormone Levels

**DOI:** 10.1371/journal.pone.0107244

**Published:** 2014-09-11

**Authors:** Márton Doleschall, Julianna Anna Szabó, Júlia Pázmándi, Ágnes Szilágyi, Klára Koncz, Henriette Farkas, Miklós Tóth, Péter Igaz, Edit Gláz, Zoltán Prohászka, Márta Korbonits, Károly Rácz, George Füst, Attila Patócs

**Affiliations:** 1 3rd Department of Internal Medicine, Semmelweis University, Budapest, Hungary; 2 Molecular Medicine Research Group, Hungarian Academy of Sciences and Semmelweis University, Budapest, Hungary; 3 William Harvey Research Institute, Barts and The London School of Medicine and Dentistry, Queen Mary University of London, London, United Kingdom; 4 2nd Department of Internal Medicine, Semmelweis University, Budapest, Hungary; 5 “Lendület” Hereditary Endocrine Tumours Research Group, Hungarian Academy of Sciences and Semmelweis University, Budapest, Hungary; University of Tennessee, United States of America

## Abstract

**Purpose:**

Systematic evaluation of the potential relationship between the common genetic variants of *CYP21A2* and hormone levels.

**Methods:**

The relationships of *CYP21A2* intron 2 polymorphisms and haplotypes with diverse baseline and stimulated blood hormone levels were studied in 106 subjects with non-functioning adrenal incidentaloma (NFAI). The rationale for using NFAI subjects is dual: i) their baseline hormone profiles do not differ from those of healthy subjects and ii) hormone levels after stimulation tests are available.

**Results:**

The carriers (N = 27) of a well-defined *CYP21A2* haplotype cluster (c5) had significantly elevated levels of cortisol (p = 0.0110), and 17-hydroxyprogesterone (p = 0.0001) after ACTH stimulation, and 11-deoxycortisol after metyrapone administration (p = 0.0017), but the hormone values were in normal ranges. In addition, the carriers (N = 33) of the C allele of the rs6462 polymorphism had a higher baseline aldosterone level (p = 0.0006). The prevalence of these genetic variants of *CYP21A2* did not differ between NFAI and healthy subjects.

**Conclusions:**

The common *CYP21A2* variants presumably exert the same effect on hormone levels in the healthy and disease-affected populations. Therefore, they may contribute to complex diseases such as some cardiovascular diseases, and may influence the genotype-phenotype correlation in patients with congenital adrenal hyperplasia (CAH) including the individual need for hormone substitution.

## Introduction

Human steroid 21-hydroxylase enzyme (cytochrome P450c21) is expressed uniquely in the adrenal cortex, catalyzes the 21-hydroxylation of progesterone to 11-deoxycorticosterone and 17-hydroxyprogesterone (17-OHP) to 11-deoxycortisol in the biosynthesis of mineralocorticoids (aldosterone) and glucocorticoids (cortisol) [1], and is encoded by the *CYP21A2* gene (OMIM: 613815) [2]. *CYP21A2* is located together with the complement component 4A and 4B genes (*C4A* and *C4B*) in a multi-allelic, complex and tandem copy number variation (CNV), called RCCX CNV, and, in a wider sense, in the major histocompatibility complex of chromosome 6 [3], [4]. RCCX CNV also comprises the duplicated non-functional pseudogene of steroid 21-hydroxylase (*CYP21A1P*). In addition to the single copy of *CYP21A2*, zero, one or two copies of *CYP21A1P* can reside in one chromosome, depending on the copy number of the repeated region in RCCX CNV [5]. Rarely, there is no *CYP21A2*, or there is more than one *CYP21A2* copy on one chromosome, usually resulting in one or three gene copies in the diploid state [6], [7]. In the majority of cases, classical congenital adrenal hyperplasia (CAH, OMIM: 201910) is caused by the complete lack of functional *CYP21A2* (CAH is divided in two main forms; a classical one with severe symptoms and a non-classical one with milder symptoms) [8].

Adrenal incidentalomas have become a rather common finding in clinical practice since the widespread introduction of abdominal imaging procedures such as computed tomography (CT) and ultrasonography for non-endocrinological reasons. The current prevalence of unsuspected adrenal tumors range between 1% and 6% [9]. Pheochromocytomas, adrenocortical carcinomas and adrenocortical hormone overproduction syndromes (including subclinical cortisol hypersecretion described as subclinical Cushing’s syndrome) are relatively rare, and the vast majority of adrenal tumors are benign non-functioning adrenal incidentalomas (NFAIs) [10], [11]. NFAI confers a slightly elevated risk of malignancy, but the hormone profiles of subjects with NFAI do not differ from those in subjects without adrenal lesions [12]. There are numerous hypotheses about the development of NFAI including, among others, the contribution of *CYP21A2*. An elevated 17-OHP response to adrenocorticotrophic hormone (ACTH) administration, which is a characteristic of CAH, has been also observed in approximately half of the subjects with NFAI [13], [14]. Consistent with these data, the heterozygote carriers of loss-of-function mutations of *CYP21A2* occurred more frequently in a study involving 50 NFAI subjects [15]. However, the role played by *CYP21A2* in the epidemiology and pathogenesis of NFAI is controversial, and the potential mechanism is unexplored [16].

In spite of recent advances in the discovery of CNVs [17], the complex, multi-allelic CNVs and their gene contents such as RCCX CNV and *CYP21A2* are underexplored due to technical difficulties [18]. While numerous studies have explored *CYP21A2* in the context of CAH, little information is available on the genetic variants of *CYP21A2* in correlation with circulating hormone levels in healthy or NFAI subjects. Variants of the full-length *CYP21A2* haplotype have been recently determined largely by molecular haplotyping [4]. Furthermore, *CYP21A2* intron 2 has been proven to be under positive selection in a healthy European population [19], implying that this subregion can exert an influence on steroid hormone levels. Moreover, the gene copy number of *C4B* (a genetic element of RCCX CNV) is associated with hormone levels in subjects with NFAI [20].

The primary aim of the current study is to reveal the putative relationship of common *CYP21A2* haplotypes (and polymorphisms) with baseline and stimulated hormone levels (the direct and causal relationship between *CYP21A2* and its metabolites is highly probable). We investigated the haplotypes of *CYP21A2* intron 2 and the 5′-end of exon 3 as “complex genetic markers” [21], because they characterize well the full-length *CYP21A2* haplotypes [19]. A population of subjects with NFAI was applied to this, because the baseline hormone profiles of NFAI subjects do not differ from those of healthy subjects, but the NFAI subjects had wider available hormone profiles including hormone levels after ACTH and metyrapone administrations (such administrations to healthy subjects conflict with medical ethics [22]). In addition, the genetic differences between the NFAI and healthy control subjects were also investigated (we did not examine the hormone levels of healthy subjects, therefore, there was no case-control approach from the viewpoint of hormone levels), with the secondary aim of evaluating the putative predisposing and protecting *CYP21A2* variant(s) for NFAI using the allele frequencies of genetic variants.

## Materials and Methods

### Subjects

A total of 125 subjects with adrenal incidentaloma diagnosed by abdominal CT or ultrasonography were enrolled into this retrospective study at the 2nd Department of Internal Medicine, Semmelweis University (Budapest, Hungary) (Table 1). All NFAI subjects had European origin, and were genetically unrelated. The presence of clinically overt hormone production, aldosterone-secreting and cortisol-producing tumors (including subclinical Cushing’s syndrome), pheochromocytoma and adrenal carcinoma, and adrenal surgery were exclusion criteria. In addition, 68 healthy control subjects (female: 65.2%, median age: 37.5, interquartile range of age: 31–43), without any clinical signs of hormone overproduction or metabolic disorders, were enrolled. The study protocol was approved by the Hungarian Health Science Council (84-358/2008-1018EKU) and by the Institutional Ethics Committee of Semmelweis University. All subjects gave written informed consent according to the Declaration of Helsinki [22].

**Table 1:** Characteristics of Hungarian subjects with non-functioning adrenal incidentaloma before genetic exclusions.

	All subjects (N = 125)
Female (%)	76.0
Age (year)	53 (46.5–61)
BMI (kg/m^2^)	29.2 (25.8–32.4)
Diameter of adenoma (mm)	23.5 (15.0–32.0)
ACTH (pmol/l)	3.82 (2.60–5.29)
metyrapone-induced ACTH (pmol/l)	40.3 (15.5–63.6)
DHEA-S (µmol/l)	3.94 (2.47–7.13)
morning cortisol (nmol/l)	340 (276–421)
midnight cortisol (nmol/l)	83 (55–138)
LDDST cortisol (nmol/l)	55 (28–74)
Hypertension (%)	78.8
DM 2 (%)	32.0
CAH mutation carriers (%)	9.6^a^
<2 *CYP21A2* gene copies (%)	5.6^b^

BMI – body mass index, ACTH – adrenocorticotrophic hormone, DHEA-S – dehydroepiandrosterone sulfate, LDDST – low dose dexamethasone test, DM 2– type 2 diabetes mellitus, CAH – congenital adrenal hyperplasia and *CYP21A2*– human steroid 21-hydroxlylase gene. Values are medians except for percentage values, and interquartile ranges are in parentheses.

a There was no significant difference in prevalence (Fisher’s exact test: p = 1.0000) compared to healthy Hungarian control subjects.

b There was no significant difference in prevalence (Fisher’s exact test: p = 0.5516) compared to healthy Hungarian control subjects.

### Hormone measurements

Hormonal evaluation included morning (at 0800–0900 h) serum cortisol, corticosterone, 17-OHP, plasma ACTH, aldosterone and midnight (at 2400 h) cortisol. Serum cortisol, corticosterone, 17-OHP and plasma aldosterone concentrations were measured after ACTH administration (a single dose of Cortrosyn or Synacthen depot, 1 mg im), while serum cortisol level was also determined after a 1 mg dexamethasone suppression test. Plasma ACTH and 11-deoxycortisol levels were measured after metyrapone administration (a single dose, 30 mg/kg bodyweight, orally). Plasma ACTH and serum cortisol concentrations were measured by electrochemoluminescent immunoassays (Elecsys, Cobas E411, Roche, Basel, Switzerland). Plasma aldosterone, serum corticosterone, dehydroepiandrosterone sulfate (DHEA-S), 11-deoxycortisol and 17-OHP levels were determined with radioimmunoassays [14], and polyclonal antibodies for these measurements were the generous gifts from professor Paul Vecsey (Institute of Pharmacology, University of Heidelberg, Germany). Plasma renin activity was determined under resting conditions using a commercially available kit (Rianen, DuPont, Boston, MA, USA).

### Genetic analyses

The gene copy numbers of *C4* and *CYP21A2*, and the haplotypes of intron 2 and the 5′-part of exon 3 of *CYP21A2* (the region will be henceforth denoted as ‘*CYP21A2* intron 2′) were determined as we have previously described [4], [19], [23]. Briefly, *C4*-type-specific quantitative PCRs (qPCR) [23], and *CYP21*-gene-specific qPCR [19] were applied to determine gene copy numbers. Haplotypic or genotypic nested polymerase chain reaction (PCR) products of *CYP21A2* were generated from the templates of allele-specific long-range PCRs [4], then *CYP21A2* intron 2 was sequenced by SEQ_12F_v2 and SEQ_16R sequencing primers [19]. In addition to the CAH mutations detected by the aforementioned methods, the six commonly occurring CAH mutations (I172N, exon 6 cluster, p.V281L, p.L307insT, p.Q318* and p.R356W) were also determined [24]. Experimentally indeterminable haplotypic *CYP21A2* intron 2 sequences were resolved by the PHASE software v2.1.1 [25], [26]. A phylogenetic tree of the *CYP21A2* intron 2 haplotype variants and Hardy-Weinberg equilibrium (HWE) were calculated by Arlequin v3.5 [27]. Linkage disequilibrium (LD) was evaluated using DnaSP v5.10.01 [28], and was visualized by Haploview v4.2 [29].

### Statistical analyses

To find the relationship between hormone levels and *CYP21A2* intron 2 haplotypes, the haplotype tree of *CYP21A2* intron 2 was scanned by TreeScan v1.0 [30]. Traditional statistical analyses were performed with G*Power v3.1.3 [31], SPSS v20 (SPSS Inc., Chicago, IL, USA) and STATISTICA v8 (Statsoft inc. Tulsa, OK, USA) software. Briefly, the outliers of hormone data were filtered based on a two-sided standard deviation, and the normal distributions of hormone datasets were tested by the Kolmogorov-Smirnov test. The datasets passing the normality test were analysed by unpaired t-test and analysis of variance (ANOVA), and the datasets with non-normal distribution were examined by Mann-Whitney and Kruskal-Wallis tests. The genetic associations adjusted for gender, age, tumor diameter and body mass index (BMI) and correlations between steroid hormone levels and adjusting variables were calculated by multiple logistic regression analysis with cutoff values obtained by receiver operating characteristic (ROC) analysis. All tests were two-tailed.

## Results

### Genetic exclusions and the validity of genetic data

In 125 enrolled subjects with NFAI (Table 1), 12 subjects had *CYP21A2* CAH mutations in a carrier state (7 of 12 subjects carried a p.V281L mutation, which causes non-classical CAH in homozygous form) and 7 subjects with more than two *CYP21A2* gene copies were observed. These 19 subjects were excluded from further analyses of the current study in order to avoid the potential disturbing effect of these genetic variants on hormone levels. In 212 chromosomes of the remaining 106 subjects, 116 (54.7%) *CYP21A2* intron 2 haplotypes were experimentally determined (by molecular haplotyping), and 88 (41.5%) haplotypes were resolved *in silico*. Eight (3.8%) haplotypes of four subjects fell below the confidence probability threshold of *in silico* reconstruction, and were unresolved. Because the combined molecular and inferred haplotyping approach ensures highly reliable results [4], all resolved haplotypes were regarded as valid, but the four subjects carrying unresolved haplotypes were excluded, and therefore 102 subjects were included in the haplotype-based analyses. The healthy control subjects went through the same genetic exclusion process, resulting in 54 studied subjects (7 subjects harbored CAH mutations in a carrier state, 6 subjects had more than two *CYP21A2* gene copies, and *CYP21A2* haplotypes were unresolved in 1 subject out of 68 enrolled healthy subjects. Six out of 7 subjects with CAH mutations carried the non-classical p.V281L mutation.). There were no significant differences in either the prevalence of CAH mutation carriers (Fisher’s exact test: p = 1.0000) or the prevalence of the individuals with more than two *CYP21A2* gene copies (Fisher’s exact test: p = 0.5516) between the populations of NFAI and healthy control subjects. Overall, 26 kinds of *CYP21A2* intron 2 haplotype were observed in NFAI subjects (Table S1), 4 kinds were unique for the NFAI subjects, and 8 kinds did not occur or were excluded in a previous exclusion step due to CAH mutations in the NFAI subjects, but were observed in recent studies [4], [19]. The 26 different *CYP21A2* intron 2 haplotype variants adequately marked the full-length *CYP21A2* haplotype variants (Table S1), and the haplotypes did not deviate from HWE in either population (NFAI: p = 0.0595 control: p = 0.2234). There were no significant differences in haplotype distributions (Chi^2^ test: p = 0.9471) and in the prevalence of alleles of *CYP21A2* intron 2 polymorphisms between the subjects with NFAI and the healthy control subjects (Fisher’s exact test: p = 0.3129–1.000, Chi^2^ test of site 605 (rs6451): p = 0.2505, the polymorphisms (sites) are numbered from the start of the *CYP21A2* coding region in the sequence of NT_007592.15: 31945792-31949720).

### Searching for the relationship between hormone levels and *CYP21A2* intron 2 haplotypes

**Figure 1:**
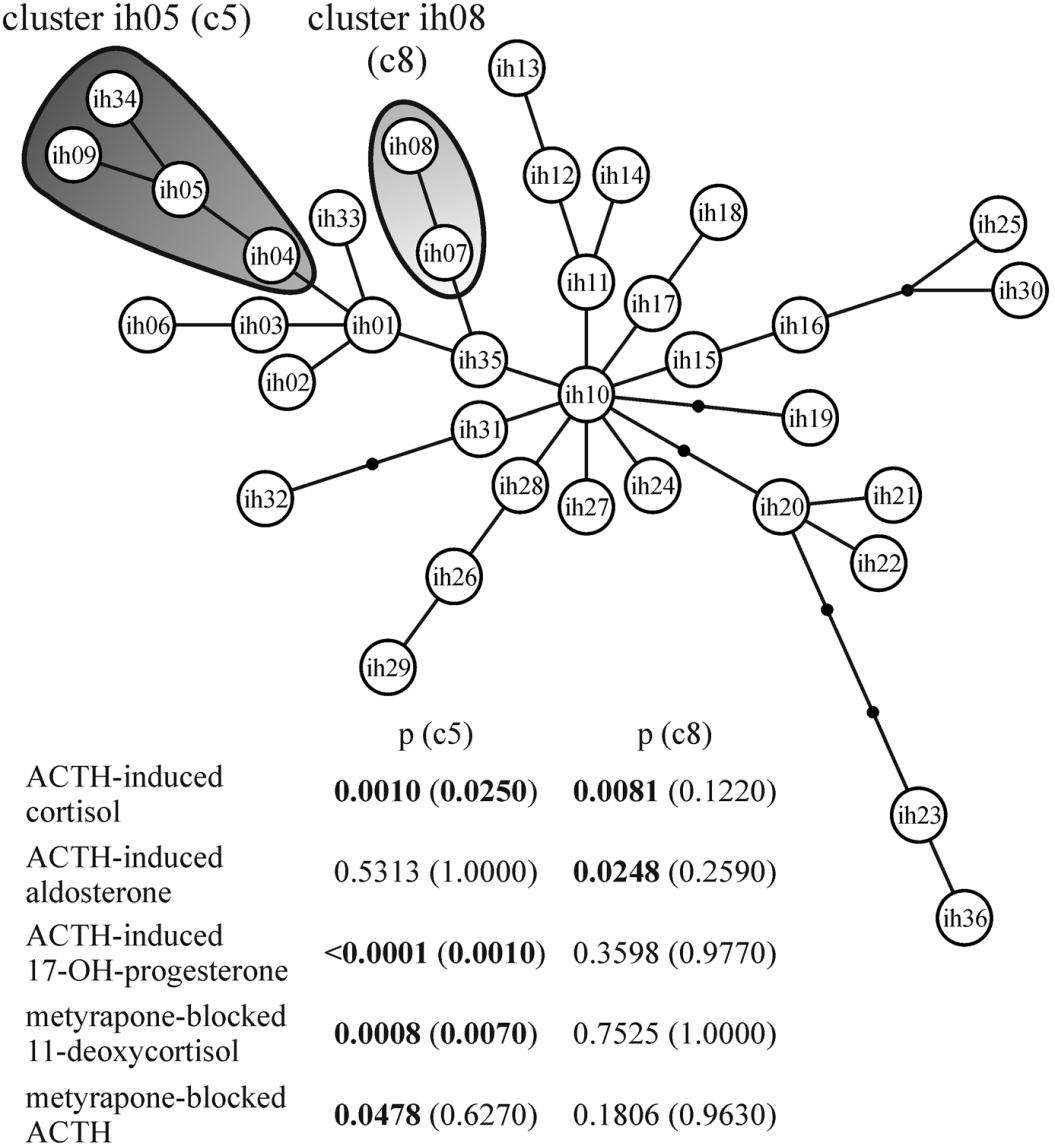
Haplotype tree of *CYP21A2* intron 2 and the 5′-part of exon 3, and the *CYP21A2* haplotype clusters with multiple significances found by tree scanning, and p-values of the haplotype clusters related to different steroid hormone levels in subjects with non-functioning adrenal incidentaloma. Circles represent the *CYP21A2* intron 2 and the 5′-part of exon 3 haplotypes, lines with or without small black circles indicate the allele differences (character-state changes) between haplotype variants and gray shapes encompass the haplotype clusters with multiple significances. The values without parentheses are uncorrected p-values, and the values with parentheses are the corrected step-down permutational p-value after enforcing monotonicity. ACTH – adrenocorticotrophic hormone.

The *CYP21A2* intron 2 haplotypes or the clusters of the haplotypes having different hormone levels than other haplotypes or clusters were searched by a tree scanning method in subjects with NFAI (N = 102). Briefly, this method splits the phylogenetic tree of *CYP21A2* intron 2 haplotypes into two parts in all possible ways, and serially tests the differences of hormone levels of NFAI subjects between the two parts. Two clusters of evolutionary-related *CYP21A2* intron 2 haplotypes (sharing highly similar allele compositions inside the clusters) showed multiple significances in the steroid hormone levels (Figure 1). The cluster of ih04, ih05, ih09 and ih34 haplotypes named c5 for the most prevalent haplotype of the cluster (ih05) significantly differed in ACTH-induced cortisol, ACTH-induced 17-OHP and metyrapone-blocked 11-deoxycortisol levels. The c5 haplotypes occurred with 14.2% prevalence in the *CYP21A2* intron 2 haplotypes of subjects with NFAI, and this prevalence did not deviate (Fisher’s exact test: p = 0.4240) from the prevalence (18.5%) observed in the healthy population (N = 54). The cluster of ih07 and ih08 haplotypes termed c8 significantly differed in ACTH-induced cortisol and ACTH-induced aldosterone levels. The c8 haplotypes occurred with an 8.8% prevalence in subjects with NFAI, and it also did not significantly deviate (Fisher’s exact test: p = 0.8308) from that of the healthy population (7.4%).

### Relationship between hormone levels and the *CYP21A2* intron 2 haplotype clusters

**Figure 2:**
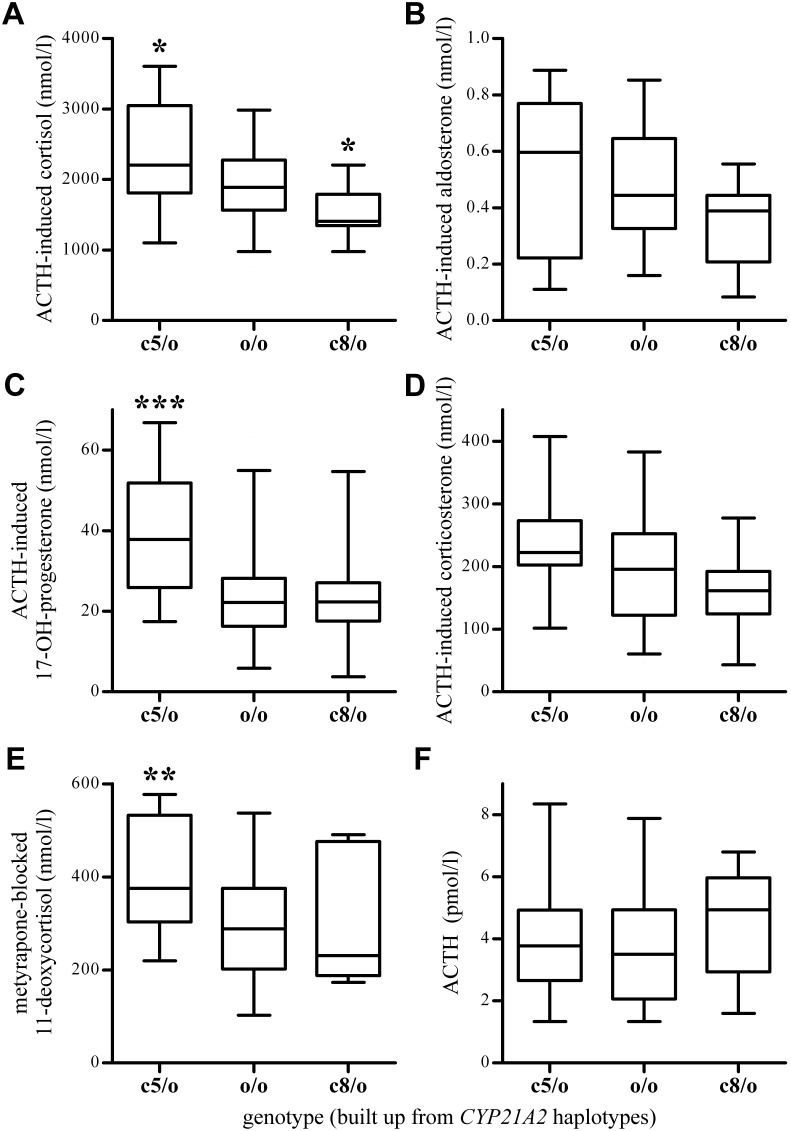
Concentrations of hormones in blood decomposed by *CYP21A2* intron 2 haplotype carrier groups in subjects with non-functioning adrenal incidentaloma. c5/o and c8/o indicate the carriers (heterozygotes) of c5 and c8 haplotypes, respectively, and o/o abbreviates the genotypes of other haplotypes. Boxes indicate interquartile ranges, lines in boxes show the medians, whiskers represent the 5^th^–95^th^ percentiles, asterisks above the boxes indicate the significant differences between the hormone levels of genotypes. One, two and three asterisks indicate p<0.05, p<0.01 and p<0.001 respectively (t-test or Mann-Whitney test). A) Serum cortisol level after adrenocorticotropic hormone (ACTH) stimulation. B) Serum aldosterone level after ACTH stimulation. C) Serum 17-OH-progesterone level after ACTH stimulation. D) Serum corticosterone level after ACTH stimulation. E) Serum 11-deoxycortisol level after metyrapone administration. F) Baseline ACTH level.

To further investigate the relationship between hormone levels and the revealed haplotype clusters, the subjects with NFAI were divided into three genotype groups based on the results of tree scanning (Table 2); the carriers of c5 haplotypes (N = 27), the carriers of c8 haplotypes (N = 16) and the NFAI subjects carrying neither of these two haplotype clusters (N = 57). Only two NFAI subjects were carriers of both haplotype clusters, necessitating their exclusion from statistical analysis. The majority of the hormone values were far inside normal ranges; however, some of them were close to the boundaries of normal ranges [32]. The baseline morning levels of cortisol, 17-OHP, corticosterone, DHEA-S, ACTH and midnight level of cortisol did not differ significantly between the genotype groups. In contrast, significant differences were found in morning aldosterone, ACTH-induced cortisol, ACTH-induced 17-OHP, metyrapone-blocked 11-deoxycortisol, and metyrapone-blocked ACTH levels. However, the subsequent statistical power analyses (the probability of correctly rejecting a false null hypothesis [33]) of these tests supported reliable (above 0.8 power) differences only in the ACTH-induced cortisol, ACTH-induced 17-OHP and metyrapone-blocked 11-deoxycortisol levels (Table 2). There was an obvious tendency to differences in ACTH-induced steroid hormone levels between the genotype groups (Figure 2), and the multivariate ANOVA test of normalized ACTH-induced cortisol, ACTH-induced aldosterone and ACTH-induced corticosterone levels showed a significant difference (p = 0.0213, power = 0.8358).

**Table 2:** Relationship between hormone levels in blood and *CYP21A2* haplotype carrier groups in subjects with non-functioning adrenal incidentaloma.

	p (K-S)	c5/o, N = 27 (median, 25^th^–75th percentile)	o/o, N = 57 (median, 25^th^–75th percentile)	c8/o N = 16 (median, 25^th^–75th percentile)	p (between allgroups,ANOVA or K-W)	p (between c5/oand o/ogenotypes, t-testor M-W)	p (between c8/oand o/ogenotypes, t-testor M-W)
cortisol (morning, nmol/l)	ns	352 (303–411)	313 (248–428)	331 (276–414)	0.8363	0.6654	0.7983
cortisol (midnight, nmol/l)	<0.05	83 (55–137)	83 (55–137)	66 (39–83)	0.2858	0.5333	0.1978
cortisol (ACTH-induced, nmol/l)	ns	**2207** (1824–3035)	**1889** (1568–2271)	**1407** (1368–1694)	**0.0006** a (**0.9161**)	**0.0110** (0.7123)	**0.0188** (0.4488)
aldosterone (morning, nmol/l)	ns	0.179 (0.097–0.277)	0.139 (0.083–0.194)	0.139 (0.090–0.196)	0.1165	**0.0488** (0.4943)	0.9383
aldosterone(ACTH-induced, nmol/l)	ns	0.596 (0.222–0.763)	0.444 (0.333–0.638)	0.388 (0.250–0.388)	0.1604	0.5744	0.0694
17-OH-progesterone (morning, nmol/l)	ns	1.48 (0.97–1.76)	1.60 (1.08–2.50)	1.76 (0.88–2.69)	0.4062	0.1935	0.5702
17-OH-progesterone (ACTH-induced, nmol/l)	<0.05	**37.8** (25.9–51.0)	**22.2** (16.4–28.2)	**22.3** (17.6–26.3)	**0.0001** (**0.9360** b)	**0.0001** (**0.9715**)	0.9431
corticosterone (morning, nmol/l)	<0.05	8.18 (5.89–10.69)	6.03 (3.90–12.53)	6.42 (2.77–11.34)	0.6173	0.3957	0.6469
corticosterone (ACTH-induced, nmol/l)	ns	222 (202–264)	195 (45–251)	161 (127–191)	0.0792	0.1236	0.2758
11-deoxycortisol (metyrapone-blocked, nmol/l)	ns	**375** (317–520)	**289** (202–375)	231 (202–462)	**0.0077** c (0.7505)	**0.0017** (**0.8372**)	0.7462
dehydroepiandro-sterone sulfate (morning, µmol/l)	<0.01	3.58 (2.47–6.45)	4.55 (2.06–6.99)	3.33 (2.85–27.05)	0.9331	0.9662	0.7742
ACTH (pmol/l)	ns	3.77 (2.68–4.91)	3.50 (2.06–4.93)	4.93 (3.02–5.84)	0.3257	0.7504	0.1475
ACTH (metyrapone-blocked, pmol/l)	ns	51.7 (33.5–76.9)	29.9 (8.5–50.0)	55.8 (39.4–87.4)	**0.0411** d (0.6013)	**0.0268** (0.5880)	0.0702

c5/o and c8/0 indicate the carriers (heterozygotes) of *CYP21A2* haplotypes of cluster ih5 and cluster ih8, respectively, o/o abbreviates the genotypes of other haplotypes, and N indicates the number of subjects. K-S – Kolmogorov-Smirnov test, ANOVA – analysis of variance, K-W – Kruskal-Wallis test, M-W – Mann-Whitney test. The normalities of hormone datasets were checked by the K-S test; the datasets passing the normality test were investigated by ANOVA and t-test; the datasets not passing were examined by M-W and K-W tests. Median values are represented, interquartile ranges are shown in parentheses below the median values, ns means non-significant result, and significant values (p<0.05) with high power (power >0.8) are highlighted in bold characters. ACTH – adrenocorticotrophic hormone.

a p values of the Newman-Keuls post-hoc test were 0.0263 between c5/o and o/o, 0.0411 between c8/o and o/o and 0.0002 between c5/o and c8/o.

b Power was calculated by simulation.

c p values of the Newman-Keuls post-hoc test were 0.0814 between c5/o and o/o, 0.7173 between c8/o and o/o and 0.0729 between c5/o and c8/o.

d p values of the Newman-Keuls post-hoc test were 0.0958 between c5/o and o/o, 0.1610 between c8/o and o/o and 0.8720 between c5/o and c8/o.

### Relationships between hormone levels, haplotype clusters and discrete *CYP21A2* intron 2 polymorphisms

Because haplotypes consist of alleles of polymorphisms, we expected that there should be some relationships among hormone levels and the discrete polymorphisms of *CYP21A2* intron 2. Moreover, the genotypes of the polymorphisms were devoid of the *in silico* haplotype reconstruction, and were completely determined by experiments. The relationships between the hormone levels and the genotypes of 10 *CYP21A2* intron 2 polymorphisms, which were not in complete LD with each other (Figure S1) and also had minor alleles occurring in more than four subjects, were investigated in the subjects with NFAI (N = 106). Only two polymorphisms, site 398 (g398, rs6462) and site 568 (g568, rs41315224) showed significant differences (p<0.05) and high power (power>0.8) simultaneously (Table S2). The prevalence of the genotypes of g398 and the prevalence of genotypes of g568 did not differ (Chi^2^ test of g398: p = 0.8736 and Chi^2^ test of g568: p = 0.0874) between the subjects with NFAI and the healthy control subjects (N = 55), and neither of polymorphisms deviated from HWE (g398: p = 0.7560 g568: p = 0.1101) in NFAI subjects. The deletion allele of g568 (g568del) was related to higher levels of ACTH-induced cortisol, ACTH-induced 17-OHP and metyrapone-blocked 11-deoxycortisol (Table S3). The occurrence of the alleles of g568 followed the c5 and c8 haplotype clusters; all c5 haplotypes carried only the g568del allele (LD between c5 haplotypes and g568del: r^2^ = 0.679), and all c8 haplotypes carried only the g568G allele (r^2^ = 0.396). In addition, the genotypes grouped by c5 and c8 haplotypes were strongly linked to the genotypes of g568 (Chi^2^ test, p<0.0001) indicating the c5 and c8 haplotypes and g568 alleles described the same genetic relationship. The g398C allele carriers were accompanied by higher morning aldosterone levels (Table S3), but neither c5 haplotypes nor c8 haplotypes harbored this allele (Table S1), and the genotypes grouped by c5 and c8 haplotypes were not related to the genotypes of g398 (Chi^2^ test p = 0.4828). Furthermore, the g398C allele occurred in many different haplotypes, and thus this genetic relationship could not be well described by *CYP21A2* intron 2 haplotypes.

### Relationships between the hormone levels, the haplotype clusters, the g398 polymorphism and other characteristics

We evaluated, using multiple logistic regression analysis, whether gender, age, diameter of adenomas and BMI besides c5 haplotypes, c8 haplotypes or g398C allele might influence steroid hormone levels proved to be significant (p<0.05) and reliable (power>0.8). The analyses did not reveal any significant relationship between steroid hormone levels and gender, age, diameter of adenomas or BMI except for the association between morning aldosterone level and age (Table 3); however, this negative correlation has already been desribed [34]. The relationships between c5 carriers and ACTH-induced cortisol, ACTH-induced 17-OHP and metyrapone-blocked 11-deoxycortisol levels, and the relationship between g398C carriers and morning aldosterone level all remained significant after adjustment for gender, age, diameter or BMI, but the relationship between c8 carriers and ACTH-induced cortisol level slightly exceeded the significance threshold (p = 0.0690).

**Table 3:** Odds ratios of gender, age, diameter of adenoma, body mass index in addition to c5 haplotypes, c8 haplotypes or g398C allele from multiple logistic regression models.

	c5 haplotypescarriers	c8 haplotypescarriers	g398C allelecarriers	Gender	age	diameter ofadenomas	BMI
ACTH-induced cortisol by ROC analysis with c5 haplotypes	**7.20** (1.49–34.9)	**-**	-	0.97 (0.24–3.87)	1.05 (0.99–1.11)	1.00 (0.96–1.05)	1.01 (0.90–1.13)
ACTH-induced 17-OH-progesterone by ROCanalysis with c5 haplotypes	**7.33** (1.66–32.3)	-	-	1.02 (0.23–4.50)	1.01 (0.95–1.07)	1.00 (0.96–1.05)	0.96 (0.86–1.08)
metyrapone-blocked 11-deoxycortisol by ROC analysis with c5 haplotype	**5.74** (1.22–27.0)	-	-	4.02 (0.64–25.3)	1.01 (0.94–1.09)	1.00 (0.95–1.05)	1.00 (0.89–1.12)
ACTH-induced cortisol by ROC analysis with c8 haplotypes	**-**	7.80 (0.85–71.5)	-	1.92 (0.45–8.16)	0.95 (0.90–1.01)	0.99 (0.96–1.02)	0.96 (0.86–1.07)
morning aldosterone by ROC analysis with g398C allele	**-**	-	**4.96** (1.18–20.9)	1.96 (0.30–12.9)	**0.96** (0.83–0.99)	1.00 (0.96–1.05)	1.02 (0.90–1.16)

ACTH – adrenocorticotrophic hormone, BMI – body mass index. Numbers indicate odds ratios (ORs), and 95% confidence intervals are in parentheses. Cutoff values were obtained by receiver operating characteristic (ROC) analysis. Significant ORs p<0.05 are indicated by bold characters.

## Discussion

Two distinct *CYP21A2* intron 2 haplotype clusters, named c5 and c8, were related to the circulating steroid hormone levels in subjects with NFAI, according to the tree scanning method. Both intron 2 haplotype clusters delineate two distinct, well-defined full-length *CYP21A2* haplotype clusters [4]; five full-length haplotypes belong to c5, and three are assigned to c8 (Table S1). Therefore, the relationships between hormone levels and *CYP21A2* intron 2 haplotypes apply to full-length *CYP21A2* haplotypes.

The carriers of c5 haplotypes showed significantly increased cortisol, 17-OHP and 11-deoxycortisol responses to intrinsic (after metyrapone administration) or extrinsic (ACTH administration) elevation of ACTH levels in blood, compared to non-carriers, but hormone levels were still in the normal range. These significant findings were supported by high statistical power and significant odds ratios after adjusting for gender, age, diameter of adenomas and BMI. Moreover, the three hormonal data series were independent, further enhancing the validity of the enhanced ACTH response. We found in a previous study [14] that the NFAI patients have higher cortisol and 17-OHP responses to ACTH as healthy subjects have, but patients with subclinical Cushing’s syndrome are not excluded from this NFAI study population, and the ACTH-induced cortisol and 17-OHP levels are actually higher in the previous study than those in the current one. Cortisol and 11- deoxycortisol are downstream from the steroid 21-hydroxylase enzyme in adrenal steroidogenesis [1], and so any *CYP21A2* haplotype-related differences in enzyme activity or the gene expression level of the enzyme could directly cause the different steroid product levels. However, there is no data on the potential underlying molecular mechanism(s) which can support the functional relationship between *CYP21A2* allelic variants (haplotypes) and steroid levels. The surprising polymorphism pattern of *CYP21A2* implies that the intron 2 may affect the steroid hormone levels through gene expression [19]. The third steroid, 17-OHP, having an elevated response to ACTH in the carriers of c5 haplotypes, is the substrate of the steroid 21-hydroxylase enzyme and the enzyme is functionally unidirectional [1], hence the direct effect of *CYP21A2* haplotypes hardly explain the elevated 17-OHP response. We hypothesize that the paracrine regulation of adrenal steroidogenesis [35], either directly or indirectly activated by higher cortisol response may the mediate elevated 17-OHP response, and, in fact, an intraadrenal positive regulatory loop exerted by cortisol is observed in some cases [36].

In the c8 haplotype carriers, the significant differences in ACTH-induced cortisol level were not supported by high power and a significant adjusted odds ratio; hence we do not regard the relationship as a perfectly reliable one. In addition to this reservation, the observed subtle patterns in different hormone levels (for instance, metyrapone-blocked ACTH) might not equivocally reflect real differences in physiological conditions, but they could be derived from statistical reasons caused by the relatively low number of subjects. Aside from the exact extent of effects on different hormone levels, we consider the c5 haplotype cluster as a causal genetic variant for the enhanced ACTH response. A specific combination of some alleles harbored by c5 haplotypes is presumably responsible for the response rather than one of its alleles. To the best of our knowledge, there is no plausible explanation supporting the deviated effects of the same *CYP21A2* haplotype on hormone levels (the causal relationship between the *CYP21A2* haplotypes and steroid hormone levels is highly probable) between the NFAI and healthy populations (having no difference in hormone profile). Consequently, the elevated 17-OHP response to ACTH in some healthy subjects may render the diagnosis of non-classical CAH more difficult [37]. In addition, c5 haplotypes can potentially modify hormone levels in patients with CAH, therefore, the genotype-phenotype correlation, including the individual need for adequate hormone substitution, may be less reliably determined. The effect of c5 haplotypes on genotype-phenotype correlation may at least to some extent explain the reduced predictability of steroid 21-hydroxylase deficiency [38].

We assessed the independent effects of each *CYP21A2* intron 2 polymorphism, and the g398 and g568 polymorphisms showed significant relationships with hormone levels in the blood, supported by high statistical power. The deletion allele of the g568 polymorphism included in the c5 haplotype showed the same multiple significant associations with enhanced ACTH response as c5 haplotypes and the g568 alleles correlated closely with the genotype groups by c5 and c8 haplotypes. Therefore, g568 is not considered as an independent genetic factor. The c5 haplotypes always harbor the g568del allele in healthy subjects as well, and the genotyping of the g568 polymorphism was devoid of the *in silico* reconstruction of haplotypes, confirming the credibility of results based on the combined molecular and inferred haplotyping approach in accordance with previous confirmations [4]. Furthermore, as the g568 polymorphism resides in intron 2, it does not affect the amino acid sequence and its direct causal effect is disputable. The carriers of the g398C allele showed significantly higher morning aldosterone levels, and the high statistical power and significant odds ratio adjusted for gender, age, diameter of adenomas and BMI supported this finding. The alleles of g398 were not related to the genotype groups by c5 and c8 haplotypes, and were not assigned to any unique haplotypes or haplotype clusters, implying that this or other strongly linked *CYP21A2* polymorphism(s) in diverse *CYP21A2* haplotype variants may be causal.

In contrast to a previous finding [15], the CAH mutations in subjects with NFAI were not more prevalent than those in the healthy population, and the c5 haplotype cluster and the g398C allele were not specifically related to NFAI. Although healthy control subjects were not age-matched, and the prevalence of subjects with low gene copy number of *C4B* decreases by age [39], it is highly improbable that a putative shift in the prevalence of the c5 haplotype or g398 between the subjects with NFAI and healthy subjects could be balanced and obscured by a putative shift related to age. The c5 haplotypes constituted the most prevalent cluster in both NFAI and healthy subjects indicating that, one fourth of healthy European people may have an elevated ACTH response. In the same way, about one third of healthy Europeans may have higher baseline aldosterone levels (but still within reference range for aldosterone measurements) because of g398C or other genetically linked allele(s). Therefore, the high prevalence of the c5 haplotypes and g398C allele in the healthy population goes far beyond the direct endocrine importance, and hence the related elevated ACTH response and higher baseline aldosterone level can potentially result in diverse pleiotropic effects on complex diseases. The effects of aldosterone level on resistant hypertension [40] and on congestive heart failure [41] are well-known. The low copy number of *C4B* is associated with cardiovascular disease (CVD) morbidity and mortality [42], as well as with the elevated ACTH-induced serum cortisol level, suggesting that some genetic variants of RCCX CNV, possibly c5 haplotypes, may directly influence the individual adaptation to stress [20]. Therefore, the causal effect of c5 haplotypes on CVD morbidity and mortality throughout the elevated ACTH and stress responses is quite plausible. While a full hormonal workup of healthy volunteers with ACTH stimulation and metyrapone tests would not be ethically acceptable [22], baseline hormone measurements need to be repeated in different study populations.

## Supporting Information

Figure S1 **Linkage disequilibrium (LD) map of dimorphic polymorphisms along the intron 2 and the 5′-end of exon 3 of the** ***CYP21A2*** **gene in subjects with non-functional adrenal incidentaloma.** The polymorphisms are numbered from the start of the *CYP21A2* coding region in the sequence of NT_007592.15: 31945792–31949720, dbSNP ID are also represented. LD values are expressed in r^2^, and darker background color indicates higher LD. Polymorphism 605 (rs6451) is not represented because this visualization can handle only dimorphic polymorphisms. Some previously described polymorphisms were absent in the current dataset because their minor alleles are rare or occur only in the haplotypes which were excluded due to the harbored congenital adrenal hyperplasia mutations.
(TIF)

Table S1 **Haplotypes of** ***CYP21A2*** **intron 2 and the 5′-end of exon 3.** *CYP21A2* - steroid 21-hydroxylase gene, NFAI - non-functional adrenal incidentaloma.
(DOC)

Table S2 **Statistical significances (p-values) between hormone levels of the genotypes of discrete** ***CYP21A2*** **intron 2 polymorphisms in subjects with non-functional adrenal incidentaloma.** ACTH – adrenocorticotrophic hormone. Statistical significances of t-tests, Mann-Whitney tests, ANOVAs and Kruskal-Wallis tests were investigated in discrete *CYP21A2* intron 2 polymorphisms which were not in complete linkage disequilibrium with each other, and had minor alleles occurring in more than four subjects. Genotype groups which did not exceed 4 individuals were also not taken into account. Significant p values are followed by the values of power. Statistical significances (p<0.05) and high power (power>0.8) are highlighted with bold characters.
(DOC)

Table S3 **Relationship between hormone levels and the frequent genotypes of site 398 and site 568 in subjects with non-functional adrenal incidentaloma.** ACTH – adrenocorticotrophic hormone. CC genotype of site 398 (N = 4) and −/− genotype of site 568 (N = 1) did not exceed 4 group members, hence they were not taken into account. The normalities of hormone datasets were checked by the Kolmogorov-Smirnov (K-S) test, and the datasets passing the normality test were investigated with the t-test, whereas the datasets not passing the K-S test were examined by the Mann-Whitney test. Median values are represented, interquartile ranges are shown in parentheses below the median values, ns; non-significant result, significant hormone levels (p<0.05) with high power (power>0.8) are highlighted with bold characters.
(DOC)

## References

[1] Miller WL, Auchus RJ (2011) The molecular biology, biochemistry, and physiology of human steroidogenesis and its disorders. Endocr Rev 32: 81–151. 21051590 10.1210/er.2010-0013 PMC3365799

[2] Higashi Y, Yoshioka H, Yamane M, Gotoh O, Fujii-Kuriyama Y (1986) Complete nucleotide sequence of two steroid 21-hydroxylase genes tandemly arranged in human chromosome: a pseudogene and a genuine gene. Proc Natl Acad Sci U S A 83: 2841–2845. 3486422 10.1073/pnas.83.9.2841 PMC323402

[3] Banlaki Z, Doleschall M, Rajczy K, Fust G, Szilagyi A (2012) Fine-tuned characterization of RCCX copy number variants and their relationship with extended MHC haplotypes. Genes Immun 13: 530–535. 22785613 10.1038/gene.2012.29

[4] Banlaki Z, Szabo JA, Szilagyi A, Patocs A, Prohaszka Z, et al (2013) Intraspecific evolution of human RCCX copy number variation traced by haplotypes of the *CYP21A2* gene. Genome Biol Evol 5: 98–112. 23241443 10.1093/gbe/evs121 PMC3595039

[5] Yang Z, Mendoza AR, Welch TR, Zipf WB, Yu CY (1999) Modular variations of the human major histocompatibility complex class III genes for serine/threonine kinase RP, complement component C4, steroid 21-hydroxylase CYP21, and tenascin TNX (the RCCX module). A mechanism for gene deletions and disease associations. J Biol Chem 274: 12147–12156. 10207042 10.1074/jbc.274.17.12147

[6] Koppens PF, Hoogenboezem T, Degenhart HJ (2002) Duplication of the CYP21A2 gene complicates mutation analysis of steroid 21-hydroxylase deficiency: characteristics of three unusual haplotypes. Hum Genet 111: 405–410. 12384784 10.1007/s00439-002-0810-7

[7] Koppens PF, Smeets HJ, de Wijs IJ, Degenhart HJ (2003) Mapping of a de novo unequal crossover causing a deletion of the steroid 21-hydroxylase (CYP21A2) gene and a non-functional hybrid tenascin-X (TNXB) gene. J Med Genet 40: e53. 12746407 10.1136/jmg.40.5.e53 PMC1735462

[8] White PC, Speiser PW (2000) Congenital adrenal hyperplasia due to 21-hydroxylase deficiency. Endocr Rev 21: 245–291. 10857554 10.1210/edrv.21.3.0398

[9] Young WF Jr (2007) Clinical practice. The incidentally discovered adrenal mass. N Engl J Med 356: 601–610. 17287480 10.1056/NEJMcp065470

[10] Zeiger MA, Siegelman SS, Hamrahian AH (2011) Medical and surgical evaluation and treatment of adrenal incidentalomas. J Clin Endocrinol Metab 96: 2004–2015. 21632813 10.1210/jc.2011-0085

[11] Racz K, Pinet F, Marton T, Szende B, Glaz E, et al (1993) Expression of steroidogenic enzyme messenger ribonucleic acids and corticosteroid production in aldosterone-producing and “nonfunctioning” adrenal adenomas. J Clin Endocrinol Metab 77: 677–682. 8370688 10.1210/jcem.77.3.8370688

[12] Vassiliadi DA, Tsagarakis S (2011) Endocrine incidentalomas–challenges imposed by incidentally discovered lesions. Nat Rev Endocrinol 7: 668–680. 21709710 10.1038/nrendo.2011.92

[13] Mantero F, Terzolo M, Arnaldi G, Osella G, Masini AM, et al (2000) A survey on adrenal incidentaloma in Italy. Study Group on Adrenal Tumors of the Italian Society of Endocrinology. J Clin Endocrinol Metab 85: 637–644. 10690869 10.1210/jcem.85.2.6372

[14] Toth M, Racz K, Adleff V, Varga I, Futo L, et al (2000) Comparative analysis of plasma 17-hydroxyprogesterone and cortisol responses to ACTH in patients with various adrenal tumors before and after unilateral adrenalectomy. J Endocrinol Invest 23: 287–294. 10882146 10.1007/BF03343725

[15] Baumgartner-Parzer SM, Pauschenwein S, Waldhausl W, Polzler K, Nowotny P, et al (2002) Increased prevalence of heterozygous 21-OH germline mutations in patients with adrenal incidentalomas. Clin Endocrinol (Oxf) 56: 811–816. 12072053 10.1046/j.1365-2265.2002.01299.x

[16] Barzon L, Maffei P, Sonino N, Pilon C, Baldazzi L, et al (2007) The role of 21-hydroxylase in the pathogenesis of adrenal masses: review of the literature and focus on our own experience. J Endocrinol Invest 30: 615–623. 17848847 10.1007/BF03346358

[17] Conrad DF, Pinto D, Redon R, Feuk L, Gokcumen O, et al (2010) Origins and functional impact of copy number variation in the human genome. Nature 464: 704–712. 19812545 10.1038/nature08516 PMC3330748

[18] Abecasis GR, Auton A, Brooks LD, DePristo MA, Durbin RM, et al (2012) An integrated map of genetic variation from 1,092 human genomes. Nature 491: 56–65. 23128226 10.1038/nature11632 PMC3498066

[19] Szabo JA, Szilagyi A, Doleschall Z, Patocs A, Farkas H, et al (2013) Both positive and negative selection pressures contribute to the polymorphism pattern of the duplicated human CYP21A2 gene. PLoS One 8: e81977. 24312389 10.1371/journal.pone.0081977 PMC3843699

[20] Banlaki Z, Raizer G, Acs B, Majnik J, Doleschall M, et al (2012) ACTH-induced cortisol release is related to the copy number of the C4B gene encoding the fourth component of complement in patients with non-functional adrenal incidentaloma. Clin Endocrinol (Oxf) 76: 478–484. 21967755 10.1111/j.1365-2265.2011.04247.x

[21] Hoehe MR (2003) Haplotypes and the systematic analysis of genetic variation in genes and genomes. Pharmacogenomics 4: 547–570. 12943464 10.2217/14622416.4.5.547

[22] WMA (2013) World Medical Association Declaration of Helsinki: ethical principles for medical research involving human subjects. JAMA 310: 2191–2194. 24141714 10.1001/jama.2013.281053

[23] Szilagyi A, Blasko B, Szilassy D, Fust G, Sasvari-Szekely M, et al (2006) Real-time PCR quantification of human complement C4A and C4B genes. BMC Genet 7: 1. 16403222 10.1186/1471-2156-7-1 PMC1360677

[24] Patocs A, Toth M, Barta C, Sasvari-Szekely M, Varga I, et al (2002) Hormonal evaluation and mutation screening for steroid 21-hydroxylase deficiency in patients with unilateral and bilateral adrenal incidentalomas. Eur J Endocrinol 147: 349–355. 12213672 10.1530/eje.0.1470349

[25] Stephens M, Donnelly P (2003) A comparison of bayesian methods for haplotype reconstruction from population genotype data. Am J Hum Genet 73: 1162–1169. 14574645 10.1086/379378 PMC1180495

[26] Stephens M, Smith NJ, Donnelly P (2001) A new statistical method for haplotype reconstruction from population data. Am J Hum Genet 68: 978–989. 11254454 10.1086/319501 PMC1275651

[27] Excoffier L, Lischer HE (2010) Arlequin suite ver 3.5: a new series of programs to perform population genetics analyses under Linux and Windows. Mol Ecol Resour 10: 564–567. 21565059 10.1111/j.1755-0998.2010.02847.x

[28] Librado P, Rozas J (2009) DnaSP v5: a software for comprehensive analysis of DNA polymorphism data. Bioinformatics 25: 1451–1452. 19346325 10.1093/bioinformatics/btp187

[29] Barrett JC, Fry B, Maller J, Daly MJ (2005) Haploview: analysis and visualization of LD and haplotype maps. Bioinformatics 21: 263–265. 15297300 10.1093/bioinformatics/bth457

[30] Posada D, Maxwell TJ, Templeton AR (2005) TreeScan: a bioinformatic application to search for genotype/phenotype associations using haplotype trees. Bioinformatics 21: 2130–2132. 15681571 10.1093/bioinformatics/bti293

[31] Faul F, Erdfelder E, Lang AG, Buchner A (2007) G*Power 3: a flexible statistical power analysis program for the social, behavioral, and biomedical sciences. Behav Res Methods 39: 175–191. 17695343 10.3758/bf03193146

[32] Sereg M, Toke J, Patocs A, Varga I, Igaz P, et al (2011) Diagnostic performance of salivary cortisol and serum osteocalcin measurements in patients with overt and subclinical Cushing's syndrome. Steroids 76: 38–42. 20813120 10.1016/j.steroids.2010.08.007

[33] Balding DJ (2006) A tutorial on statistical methods for population association studies. Nat Rev Genet 7: 781–791. 16983374 10.1038/nrg1916

[34] Bauer JH (1993) Age-related changes in the renin-aldosterone system. Physiological effects and clinical implications. Drugs Aging 3: 238–245. 8324299 10.2165/00002512-199303030-00005

[35] Ehrhart-Bornstein M, Hinson JP, Bornstein SR, Scherbaum WA, Vinson GP (1998) Intraadrenal interactions in the regulation of adrenocortical steroidogenesis. Endocr Rev 19: 101–143. 9570034 10.1210/edrv.19.2.0326

[36] Lefebvre H, Prevost G, Louiset E (2013) Autocrine/paracrine regulatory mechanisms in adrenocortical neoplasms responsible for primary adrenal hypercorticism. Eur J Endocrinol 169: R115–138. 23956298 10.1530/EJE-13-0308

[37] Auchus RJ, Arlt W (2013) Approach to the patient: the adult with congenital adrenal hyperplasia. J Clin Endocrinol Metab 98: 2645–2655. 23837188 10.1210/jc.2013-1440 PMC3701266

[38] New MI, Abraham M, Gonzalez B, Dumic M, Razzaghy-Azar M, et al (2013) Genotype-phenotype correlation in 1,507 families with congenital adrenal hyperplasia owing to 21-hydroxylase deficiency. Proc Natl Acad Sci U S A 110: 2611–2616. 23359698 10.1073/pnas.1300057110 PMC3574953

[39] Kramer J, Fulop T, Rajczy K, Nguyen AT, Fust G (1991) A marked drop in the incidence of the null allele of the B gene of the fourth component of complement (C4B*Q0) in elderly subjects: C4B*Q0 as a probable negative selection factor for survival. Hum Genet 86: 595–598. 2026423 10.1007/BF00201547

[40] Calhoun DA, Jones D, Textor S, Goff DC, Murphy TP, et al (2008) Resistant hypertension: diagnosis, evaluation, and treatment: a scientific statement from the American Heart Association Professional Education Committee of the Council for High Blood Pressure Research. Circulation 117: e510–526. 18574054 10.1161/CIRCULATIONAHA.108.189141

[41] Weber KT (2001) Aldosterone in congestive heart failure. N Engl J Med 345: 1689–1697. 11759649 10.1056/NEJMra000050

[42] Arason GJ, Kramer J, Blasko B, Kolka R, Thorbjornsdottir P, et al (2007) Smoking and a complement gene polymorphism interact in promoting cardiovascular disease morbidity and mortality. Clin Exp Immunol 149: 132–138. 17425651 10.1111/j.1365-2249.2007.03391.x PMC1942025

