# Supplementary figures and images for: Common Genetic Variants of the Human Steroid 21-Hydroxylase Gene (CYP21A2) Are Related to Differences in Circulating Hormone Levels
Source: PLoS One. 2014 Sep 11;9(9):e107244. doi: 10.1371/journal.pone.0107244 (PMC4161435; doi:10.1371/journal.pone.0107244)

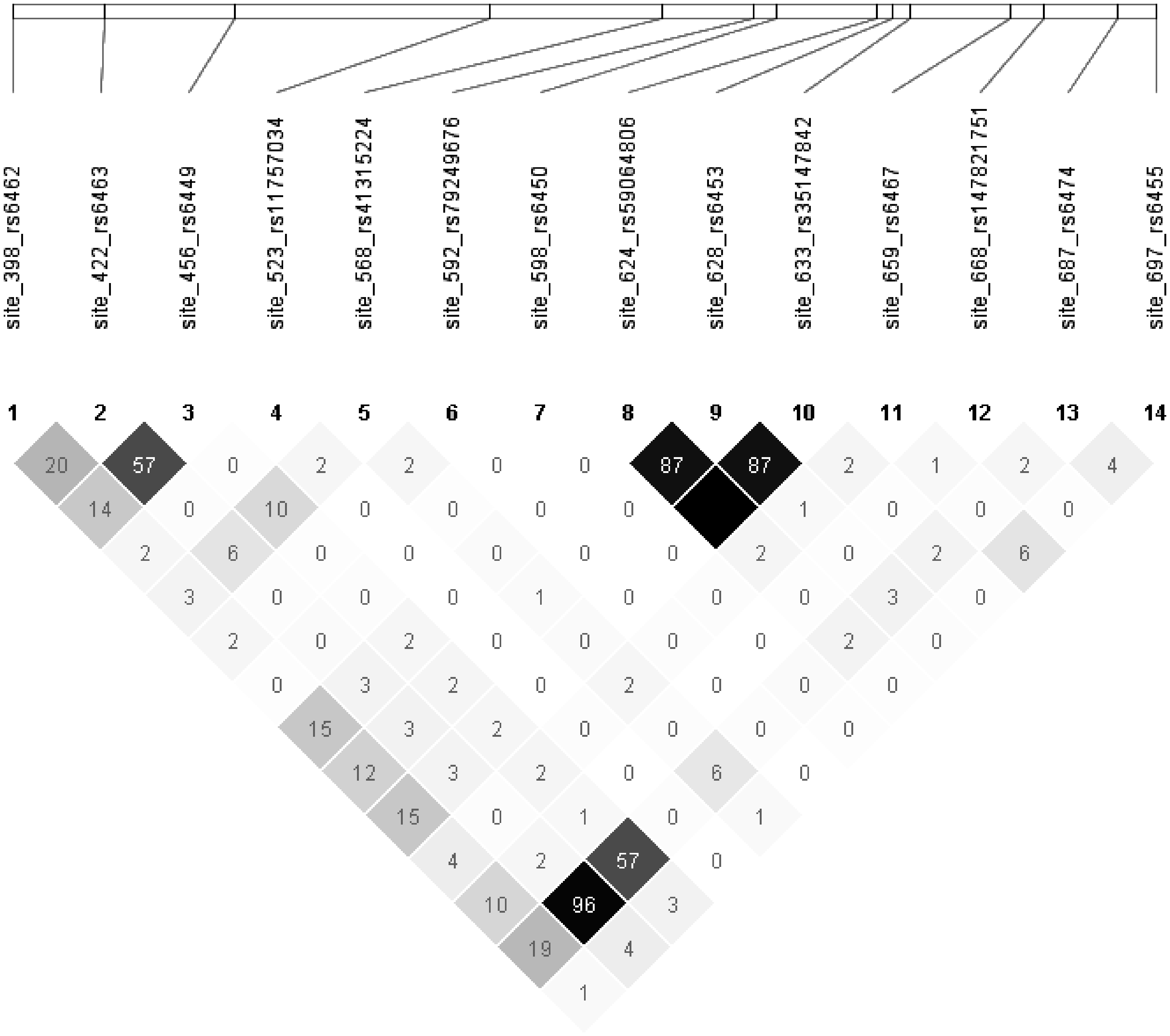

Supplement: Figure S1 — Linkage disequilibrium (LD) map of dimorphic polymorphisms along the intron 2 and the 5′-end of exon 3 of the CYP21A2 gene in subjects with non-functional adrenal incidentaloma. The polymorphisms are numbered from the start of the CYP21A2 coding region in the sequence of NT_007592.15: 31945792–31949720, dbSNP ID are also represented. LD values are expressed in r2, and darker background color indicates higher LD. Polymorphism 605 (rs6451) is not represented because this visualization can handle only dimorphic polymorphisms. Some previously described polymorphisms were absent in the current dataset because their minor alleles are rare or occur only in the haplotypes which were excluded due to the harbored congenital adrenal hyperplasia mutations. (TIF) [file pone.0107244.s001.tif]
