# Supplementary material for: Common Genetic Variants of the Human Steroid 21-Hydroxylase Gene (CYP21A2) Are Related to Differences in Circulating Hormone Levels
Source: PLoS One. 2014 Sep 11;9(9):e107244. doi: 10.1371/journal.pone.0107244 (PMC4161435; doi:10.1371/journal.pone.0107244)
Supplement: Table S1 — Haplotypes of CYP21A2 intron 2 and the 5′-end of exon 3. CYP21A2 - steroid 21-hydroxylase gene, NFAI - non-functional adrenal incidentaloma. (DOC) [file pone.0107244.s002.doc]

| *CYP21A2* intron 2 haplotype variants | full-length *CYP21A2* haplotype variants | rs6462 | rs6463 | rs184177366 | rs6449 | rs188515168 | rs192217993 | rs11757034 | rs185054992 | rs190742906 | rs41315224 | rs79249676 | rs6450 | rs6451 | rs59064806 | rs6453 | rs35147842 | rs6467 | rs147821751 | rs6474 | rs6455 | patients with NFAI | healthy control |
| --- | --- | --- | --- | --- | --- | --- | --- | --- | --- | --- | --- | --- | --- | --- | --- | --- | --- | --- | --- | --- | --- | --- | --- |
|  |  | 398 | 422 | 426 | 456 | 505 | 516 | 523 | 549 | 563 | 568 | 592 | 598 | 605 | 624 | 628 | 633 | 659 | 668 | 687 | 697 | n | n |
| ih01 | h01-09 | T | C | T | T | A | T | C | C | G | G | G | G | C | A | G | CA | A | G | A | G | 39 | 25 |
| ih02 | h10 | T | C | T | T | A | T | C | C | G | G | G | G | C | A | G | CA | C | G | A | G | 6 | 1 |
| ih03 | h11 | T | C | T | T | A | T | C | C | G | G | G | G | C | A | G | CA | G | G | A | G | 0 | 0 |
| ih04 | h12 | T | C | T | T | A | T | C | C | G | - | G | G | C | A | G | CA | A | G | A | G | 8 | 4 |
| ih05 | h13-14, h21 | T | C | T | T | A | T | C | C | G | - | G | G | C | A | G | CA | C | G | A | G | 19 | 16 |
| ih06 | h15 | T | C | T | T | A | T | C | C | G | - | G | G | C | A | G | CA | G | G | A | G | 0 | 0 |
| ih07 | h16-17 | T | C | T | C | A | T | C | C | G | G | G | G | C | A | G | CA | A | G | A | G | 9 | 3 |
| ih08 | h18 | T | C | T | C | A | T | C | C | G | G | G | G | C | A | G | CA | C | G | A | G | 9 | 5 |
| ih09 | h19 | T | C | T | C | A | T | C | C | G | - | G | G | C | A | G | CA | C | G | A | G | 1 | 0 |
| ih10 | h20, h22-25, h49 | T | A | T | C | A | T | C | C | G | G | G | G | C | A | G | CA | A | G | G | G | 5 | 3 |
| ih11 | h26-h28, h53, h60-61 | T | A | T | C | A | T | C | C | G | G | G | G | C | A | G | CA | C | G | G | G | 24 | 15 |
| ih12 | h29, h62 | T | A | T | C | A | T | C | C | G | G | G | G | C | A | G | CA | C | G | G | C | 9 | 3 |
| ih13 | h30 | T | A | T | C | A | T | C | C | G | G | G | G | C | A | T | CA | C | G | G | C | 1 | 0 |
| ih14 | h31 | T | A | T | C | A | T | C | C | G | G | G | A | C | A | G | CA | A | G | G | G | 2 | 0 |
| ih15 | h32, h55-57 | T | A | T | C | A | T | C | C | G | - | G | G | C | A | G | CA | A | G | G | G | 9 | 4 |
| ih16 | h33 | T | A | T | C | A | T | C | C | G | - | G | G | C | A | G | CA | C | G | G | G | 0 | 0 |
| ih17 | h34 | T | A | T | C | A | T | C | C | C | G | G | G | C | A | G | CA | A | G | G | G | 0 | 0 |
| ih18 | h35 | T | A | T | C | A | T | C | C | C | G | G | G | C | A | G | CA | C | G | G | G | 0 | 0 |
| ih19 | h36 | T | A | T | C | A | T | C | A | G | - | G | G | C | A | G | CA | A | G | G | G | 0 | 0 |
| ih20 | h37-38 | C | A | T | C | A | T | C | C | G | G | G | G | A | A | G | CA | A | G | G | G | 8 | 3 |
| ih21 | h39-40 | C | A | T | C | A | T | C | C | G | G | G | G | A | A | G | CA | A | A | G | G | 5 | 1 |
| ih22 | h41 | C | A | T | C | A | T | C | C | G | G | G | G | A | A | G | CA | C | G | G | G | 1 | 1 |
| ih23 | h42-43 | C | A | T | C | A | T | C | C | G | G | G | G | A | G | T | GG | A | G | G | G | 6 | 5 |
| ih24 | h44 | C | A | T | C | A | T | C | C | G | G | G | G | C | A | G | CA | C | G | G | G | 5 | 2 |
| ih25 | h45 | C | A | T | C | A | T | C | C | G | - | A | G | C | A | G | CA | C | G | G | G | 1 | 1 |
| ih26 | h46 | T | A | T | T | A | T | C | C | G | G | G | G | G | A | G | CA | A | G | G | G | 5 | 2 |
| ih27 | h47-48 | T | A | T | C | A | T | C | C | G | G | G | G | A | A | G | CA | C | G | G | G | 9 | 3 |
| ih28 | h50,h54 | T | A | T | C | A | T | C | C | G | G | G | G | G | A | G | CA | A | G | G | G | 7 | 1 |
| ih29 | h51 | T | A | A | T | A | T | C | C | G | G | G | G | G | A | G | CA | A | G | G | G | 0 | 0 |
| ih30 | h52 | C | A | T | C | A | T | T | C | G | - | G | G | C | A | G | CA | C | G | G | G | 1 | 1 |
| ih31 | h58 | C | A | T | C | A | T | C | C | G | G | G | G | C | A | G | CA | A | G | G | G | 11 | 9 |
| ih32 | h59 | C | A | T | C | G | G | C | C | G | G | G | G | C | A | G | CA | A | G | G | G | 0 | 0 |
| ih33 |  | T | C | T | T | A | T | C | C | G | G | G | G | A | A | G | CA | A | G | A | G | 1 | 0 |
| ih34 |  | T | C | T | T | A | T | C | C | G | - | G | G | C | A | G | CA | C | G | G | G | 1 | 0 |
| ih35 |  | T | C | T | C | A | T | C | C | G | G | G | G | C | A | G | CA | A | G | G | G | 1 | 0 |
| ih36 |  | C | A | T | C | A | T | C | C | G | G | G | G | C | G | T | GG | A | G | G | G | 1 | 0 |
|  |  |  |  |  |  |  |  |  |  |  |  |  |  |  |  |  |  |  |  |  |  | 204 | 108 |
|  | | | | | | | | | | | | | | | | | | | | | |  |  |
