# Supplementary material for: Common Genetic Variants of the Human Steroid 21-Hydroxylase Gene (CYP21A2) Are Related to Differences in Circulating Hormone Levels
Source: PLoS One. 2014 Sep 11;9(9):e107244. doi: 10.1371/journal.pone.0107244 (PMC4161435; doi:10.1371/journal.pone.0107244)
Supplement: Table S2 — Statistical significances (p-values) between hormone levels of the genotypes of discrete CYP21A2 intron 2 polymorphisms in subjects with non-functional adrenal incidentaloma. ACTH – adrenocorticotrophic hormone. Statistical significances of t-tests, Mann-Whitney tests, ANOVAs and Kruskal-Wallis tests were investigated in discrete CYP21A2 intron 2 polymorphisms which were not in complete linkage disequilibrium with each other, and had minor alleles occurring in more than four subjects. Genotype groups which did not exceed 4 individuals were also not taken into account. Significant p values are followed by the values of power. Statistical significances (p<0.05) and high power (power>0.8) are highlighted with bold characters. (DOC) [file pone.0107244.s003.doc]

|  | site 398 (rs6462) | site 422 (rs6463) | site 456 (rs6449) | site 568 (rs41315224) | site 605 (rs6451) | site 624 (rs59064806) | site 628 (rs6453) | site 659 (rs6467) | site 687 (rs6474) | site 697 (rs6455) |
| --- | --- | --- | --- | --- | --- | --- | --- | --- | --- | --- |
| cortisol (morning, nmol/l) | 0.5132 | 0.6114 | 0.5182 | 0.1086 | 0.7598 | 0.0692 | **0.0121** 0.6752 | 0.4813 | 0.7849 | 0.1959 |
| cortisol (midnight, nmol/l) | 0.3163 | 0.2432 | 0.7023 | **0.0080** 0.7406 | **0.0457** 0.3690 | 0.9631 | 0.7360 | 0.7027 | 0.2084 | 0.2435 |
| cortisol (ACTH-induced, nmol/l) | 0.1452 | 0.6490 | 0.3620 | **0.0001 0.9617** | 0.5407 | 0.2008 | 0.2008 | 0.0487 | 0.5760 | 0.4491 |
| aldosterone (morning, nmol/l) | **0.0006 0.9461** | 0.5247 | 0.1373 | 0.0580 | 0.2166 | **0.0221** 0.6161 | 0.1126 | 0.0878 | 0.2964 | 0.1172 |
| aldosterone (ACTH-induced, nmol/l) | 0.6971 | 0.1877 | 0.9583 | 0.2618 | 0.0740 | 0.8205 | 0.8205 | 0.5718 | 0.1676 | 0.9308 |
| 17-OH-progesterone (morning, nmol/l) | 0.8070 | 0.7047 | 0.4518 | 0.2203 | 0.3046 | 0.4860 | 0.2913 | 0.8749 | 0.2328 | 0.4928 |
| 17-OH-progesterone (ACTH-induced, nmol/l) | **0.0161** 0.6933 | 0.3737 | 0.0509 | **<0.0001 0.9848** | 0.6698 | 1.0000 | 1.0000 | 0.2232 | 0.3528 | 0.7474 |
| corticosterone (morning, nmol/l) | 0.3983 | 0.8497 | 0.9124 | 0.5630 | 0.9780 | 0.4242 | 0.1618 | 0.3729 | 0.8417 | 0.5345 |
| corticosterone (ACTH-induced, nmol/l) | 0.2599 | 0.3803 | **0.0235** 0.6770 | **0.0340** 0.5673 | 0.4302 | 0.8535 | 0.8535 | 0.2198 | 0.4060 | 0.9035 |
| 11-deoxycortisol (metyrapone-blocked, nmol/l) | 0.9953 | 0.2995 | 0.4593 | **0.0018 0.8514** | 0.6122 | 0.8565 | 0.7303 | 0.0470 | 0.3322 | 0.6115 |
| dehydroepiandrosterone sulfate (morning, µmol/l) | 0.8798 | 0.7493 | 0.9138 | 0.4678 | 0.4152 | 0.3408 | 0.7923 | 0.0997 | 0.7892 | 0.6071 |
| ACTH (pmol/l) | 0.8850 | 0.1077 | 0.4248 | 0.5972 | 0.4820 | 0.5867 | 0.5867 | 0.1271 | 0.0573 | **0.0243** 0.5992 |
| ACTH (metyrapone-blocked, pmol/l) | 0.4235 | 0.1993 | 0.6386 | 0.1262 | 0.3531 | 0.8549 | 0.6098 | 0.2892 | 0.1993 | 0.2229 |
