# Supplementary material for: Common Genetic Variants of the Human Steroid 21-Hydroxylase Gene (CYP21A2) Are Related to Differences in Circulating Hormone Levels
Source: PLoS One. 2014 Sep 11;9(9):e107244. doi: 10.1371/journal.pone.0107244 (PMC4161435; doi:10.1371/journal.pone.0107244)
Supplement: Table S3 — Relationship between hormone levels and the frequent genotypes of site 398 and site 568 in subjects with non-functional adrenal incidentaloma. ACTH – adrenocorticotrophic hormone. CC genotype of site 398 (N = 4) and −/− genotype of site 568 (N = 1) did not exceed 4 group members, hence they were not taken into account. The normalities of hormone datasets were checked by the Kolmogorov-Smirnov (K-S) test, and the datasets passing the normality test were investigated with the t-test, whereas the datasets not passing the K-S test were examined by the Mann-Whitney test. Median values are represented, interquartile ranges are shown in parentheses below the median values, ns; non-significant result, significant hormone levels (p<0.05) with high power (power>0.8) are highlighted with bold characters. (DOC) [file pone.0107244.s004.doc]

|  | p (K-S) | site 398 (rs6462) | | site 568 (rs41315224) | |
| --- | --- | --- | --- | --- | --- |
|  |  | CT (N=33) | TT (N=69) | -G (N=38) | GG (N=67) |
| cortisol (morning, nmol/l) | ns | 317 (276-435) | 322 (262-407) | 359 (303-441) | 303 (262-407) |
| cortisol (midnight, nmol/l) | <0.01 | 83 (55-138) | 83 (55-110) | 97 (83-166) | 66 (44-110) |
| cortisol (ACTH-induced, nmol/l) | ns | 1675 (1586-2110) | 1973 (1600-2299) | **2117** (1836-2773) | **1642** (1490-2020) |
| aldosterone (morning, nmol/l) | ns | **0.212** (0.117-0.305) | **0.139** (0.083-0.187) | 0.166 (0.097-0.277) | 0.139 (0.084-0.194) |
| aldosterone (ACTH-induced, nmol/l) | ns | 0.472 (0.319-0.583) | 0.402 (0.250-0.638) | 0.583 (0.222-0.721) | 0.416 (0.305-0.499) |
| 17-OH-progesterone (morning, nmol/l) | ns | 1.62 (1.00-2.36) | 1.50 (1.06-2.38) | 1.57 (1.06-2.15) | 1.51 (1.00-2.66) |
| 17-OH-progesterone (ACTH-induced, nmol/l) | <0.05 | 34.0 (25.1-48.6) | 23.8 (17.8-31.3) | **35.7** (25.3-51.0) | **22.2** (16.9-27.8) |
| corticosterone (morning, nmol/l) | <0.05 | 7.70 (4.44-13.31) | 6.66 (4.03-10.97) | 7.61 (4.37-11.9) | 6.41 (3.88-12.22) |
| corticosterone (ACTH-induced, nmol/l) | ns | 200 (109-219) | 202 (147-257) | 222 (186-273) | 194 (127-211) |
| 11-deoxycortisol (metyrapone-blocked, nmol/l) | ns | 303 (238-476) | 332 (202-462) | **390** (317-520) | **289** (202-375) |
| Dehydroepiandrosterone sulfate (morning, µmol/l) | <0.01 | 4.39 (2.44-7.24) | 3.70 (2.48-6.72) | 3.17 (2.41-6.45) | 4.42 (2.49-6.99) |
| ACTH (pmol/l) | ns | 3.83 (2.64-4.95) | 3.85 (2.62-5.08) | 3.42 (2.60-4.87) | 4.07 (2.47-5.13) |
| ACTH (metyrapone-blocked, pmol/l) | ns | 51.5 (32.6-70.9) | 32.3 (14.3-62.9) | 50.5 (27.4-76.6) | 32.6 (12.0-55.8) |
